# Supplementary material for: BDNF in Ventrolateral Orbitofrontal Cortex to Dorsolateral Striatum Circuit Moderates Alcohol Consumption and Gates Alcohol Habit
Source: bioRxiv. 2025 Jan 13:2025.01.09.632255. Preprint. [Version 1] doi: 10.1101/2025.01.09.632255 (PMC11761066; doi:10.1101/2025.01.09.632255)
Supplement: Supplement 1 [file media-1.docx]

**Supplementary methods**

**Reagents**

Ethyl alcohol (190 proof) was purchased from VWR (Radnor, PA), and sucrose was purchased from Fisher Scientific (Pittsburgh, PA). The Qiazol RNA isolation kit was purchased from Qiagen (Redwood City, CA). cDNA was synthesized using the iScript cDNA Synthesis Kit (BioRad). Powerup SYBR Green PCR Master mix (ThermoFisher) was used for quantitative real-time PCR. Other common reagents were from Sigma Aldrich (St. Louis, MO) or Fisher Scientific (Pittsburgh, PA).

**Preparation of solutions**

Alcohol solution was prepared from absolute anhydrous alcohol (190 proof) diluted to 20% alcohol (v/v) in tap water. Sucrose solution was diluted to 0.3% sucrose (v/v) in tap water.

**Collection of brain samples for biochemical analyses**

Mice were euthanized 4 hours and 24 hours after the last drinking session (binge and withdrawal time point). Brains were removed and dissected on an ice-cold platform into 1 mm sections, and specific subregions-vlOFC, mOFC, and motor cortex (M2) were dissected based on Allen Brain Atlas.

**Quantitative real-time PCR**

RNA was isolated using the RNeasy kit, and cDNA was synthesized using the iScript cDNA Synthesis Kit according to the manufacturer’s instructions. The resulting cDNA was used for quantitative real-time PCR, using Powerup SYBR Green PCR Master mix. Thermal cycling was performed on QuantStudio 5 real-time PCR System (Thermo Fisher Scientific Inc.) using a relative calibration curve. The quantity of *BDNF* mRNA was measured and expressed relative to *GAPDH* mRNA. PCR primers used: *BDNF* Forward 5’- TGC AGG GGC ATA GAC AAA AGG-3’, *BDNF* Reverse 5’- CTT ATG AAT CGC CAG CCA ATT CTC-3’, *GAPDH* Forward 5’-CGA CTT CAA CAG CAA CTC CCA CTC TTC C-3’ and *GAPDH* Reverse 5’- TGG GTG GTC CAG GGT TTC TTA CTC CTT-3’.

**Adeno-associated viruses**

AAV2-DIO-Ef1a-BDNF-IRES-mCherry virus (AAV2-DIO-BDNF-mCherry; 3 x 10^12^ vg/ml) and AAV2-DIO-Ef1a-mCherry virus (AAV2-DIO-mCherry; 1 x 10^12^ vg/ml) were constructed in conjunction with C&M Biolabs (Richmond, CA) and were produced by the Duke University Viral Vector Core. AAV2-retrograde(retro)-Cre-GFP virus (AAVretro.hSyn.HI.eGFP-Cre.WPRE.SV40; 3x10^12^ vg/ml) was purchased from Addgene.

**Stereotaxic viral infection**

Mice were anesthetized by vaporized isoflurane and placed on a digital stereotaxic frame (David Kopf Instruments). Two holes were drilled above the site of viral injection. The injectors (stainless tubing, 33 gauges; Small Parts Inc.) were slowly lowered into the target region. The injectors were connected to 10 µl Hamilton syringes, and the infusion was controlled by an automatic pump at a rate of 0.1 µl/min. The injectors remained in place for an additional 10 minutes to allow the virus to diffuse and then were slowly removed.

For circuit-specific expression of BDNF, mice received bilateral infusion of 1 µl of AAV2-DIO-Ef1a-BDNF-IRES-mCherry virus (AAV2-DIO-BDNF-mCherry, 3x10^12^ gc/ml) per hemisphere into the vlOFC (AP: +2.2, ML: ± 1.2, DV: -2.6) or M2 (AP: +2.2, ML: ± 1.3, DV: -1.35) and 1 µl of AAVretro-Cre-GFP (3x10^12^ vg/ml) into the DLS (AP: +1.1, ML: ± 1.9, DV: -2.95) or DMS (AP +1.1, ML +/- 1.2, DV -2.95). Control animals received 1 µl of empty vector: AAV2-DIO-Ef1a-mCherry virus (AAV-DIO-mCherry ,1x10^12^ gc/ml) in the vlOFC or M2 and 1 µl of AAVretro-Cre-GFP (3x10^12^vg/ml) in the DLS or DMS.

**Confirmation of viral expression**

At the end of the experiments, animals were euthanized via cervical dislocation, the brains were removed, placed on ice, and dissected into 1 mm coronal sections. The fluorescent protein expressed by the virus (either GFP or mCherry) was visualized using an EVOS FL tabletop fluorescent microscope and images were obtained (ThermoFisher Scientific). Two animals that failed to exhibit fluorescence associated with viral overexpression were excluded from the study.

**Behavioral paradigms**

**Intermittent access to 0.3 % sucrose two-bottle choice**

Sucrose intake paradigm was conducted as previously described (Hoisington et al., 2024). Animals received one bottle of 0.3% sucrose and one bottle of water 24 hours a day for two weeks on Monday, Wednesday, and Friday, with 24 or 48 hours (weekend) sucrose deprivation periods in which mice consumed only water. Sucrose solution intake (ml/kg), water intake (ml/kg), total fluid intake (ml/kg), and the preference ratio (volume of sucrose solution intake/total volume of fluid intake) were recorded every 24 hours for the duration of sucrose access. Corrections were made to account for spillage based on bottles affixed to an empty cage.

**Open-field locomotion test**

Mice were habituated to the room for 60 minutes prior to the experiment. Mice were placed in an open field apparatus (43cm x 43cm) in low-light conditions and allowed to explore for 10 minutes ^51^. Locomotor activity was tracked using EthoVision XT software (Noldus, Leesburg, VA), and total movement (cm) and velocity (cm/s) were recorded. At the end of the session, the mouse was removed, and the apparatus was cleaned between sessions.

| **Figure** | **Average Consumption (g/kg/24hr)** | **SEM (±g/kg/24hr)** |
| --- | --- | --- |
| 1B-D and Sup. Fig 1A Males | 14.47 | 1.00 |
| 1E-F and Sup. Fig 1D Females | 19.27 | 1.10 |
| 2F AAV-DIO-mCherry | 15.07 | 1.28 |
| 2F AAV-DIO-BDNF-mCherry | 11.86 | 1.11 |
| 2I AAV-DIO-mCherry | 91.62 (ml/kg) | 9.62 (ml/kg) |
| 2I AAV-DIO-BDNF-mCherry | 101.27 (ml/kg) | 15.69 (ml/kg) |
| 3C AAV-DIO-mCherry | 15.86 | 1.54 |
| 3C AAV-DIO-BDNF-mCherry | 18.02 | 1.67 |
| 3G AAV-DIO-mCherry | 11.81 | 0.86 |
| 3G AAV-DIO-BDNF-mCherry | 14.79 | 1.87 |
| 5C TrkB agonist | 16.51 | 0.79 |
| Sup. Fig 6A AAV-DIO-mCherry | 16.56 | 1.62 |
| Sup. Fig 6A AAV-DIO-BDNF-mCherry | 18.95 | 1.73 |

**Supplementary Table S1. Average alcohol and sucrose consumption for behavior experiments**

Average alcohol and sucrose consumption in mice subjected to the IA2BC with 20% alcohol and 0.3% sucrose, respectively.

**Supplementary Figures**

**Supplementary Figure 1. Drinking profile of male and female mice**

Mice were subjected to IA20%2BC for 7 weeks in the home cage before harvesting vlOFC, mOFC and M2 regions for biochemical analysis. Alcohol consumption, preference, and water consumption of male (**a-c**) and female mice (**d-f**).

**Supplementary Figure 2. Confirmation of the Cre/DIO strategy efficacy**

(**a**) Schematic representation of BDNF overexpression in vlOFC-to-DLS circuit. Mice received bilateral injections of AAVretro-Cre-GFP in the DLS. (**b**) Representative images of GFP expression (AAVretro-Cre-GFP) in the DLS and in cortical regions, including the vlOFC. (**c**) Mice received bilateral injections of AAV2-DIO-BDNF-mCherry in vlOFC. (**d**) Representative images showing no mCherry expression following AAV2-DIO-BDNF-mCherry injections in the vlOFC without AAVretro-Cre-GFP injection in the DLS

**Supplementary Figure 3. Overexpression of BDNF in vlOFC-to-DLS projecting neurons does not alter water or total fluid consumption during alcohol and sucrose intake**

(**a-b**) Water and total consumption of alcohol was measured after BDNF overexpression in vlOFC-to-DLS projecting neurons **(a)** Water consumption was measured (Two-Way ANOVA, effect of BDNF overexpression, F _(1, 13)_ = 2.906, p =0.11, effect of session, F _(20, 245)_ = 4.437, ****p <0.0001, effect of interaction F_(20,245)_ = 0.92, p = 0.55). (**b**) Total fluid consumption was calculated (Two-Way ANOVA, effect of BDNF overexpression, F _(1, 13)_ = 1.888, p =0.19, effect of session, F _(20, 245)_ = 3.900, ****p <0.0001, effect of interaction F_(20,248)_ = 0.87, p = 0.61). (**c-d**) Water and total consumption of sucrose was measured after BDNF overexpression in vlOFC-to-DLS projecting neurons (**c**) Water consumption was measured (Two-Way ANOVA, effect of BDNF overexpression, F _(1, 11)_ = 0.2527, p =0.62, effect of session, F _(6, 58)_ = 0.8752, p =0.51, effect of interaction, F _(6, 58)_ = 2.479, p =0.03). (**d**) Total fluid consumption was calculated (Two-Way ANOVA, effect of BDNF overexpression, F _(1, 11)_ = 0.4471, p =0.51, effect of session, F _(6, 56)_ = 1.682, p =0.14, effect of interaction, F _(6, 56)_ = 2.386, p =0.04). Data are represented as mean ± SEM, n=6-9 per group

**Supplementary Figure 4. Overexpression of BDNF in vlOFC-to-DLS projecting neurons does not alter locomotion**

(**a**) Experimental timeline: Mice received bilateral injections of AAV2-DIO-BDNF-mCherry in the vlOFC and AAVretro-Cre-GFP in the DLS. Three weeks after the surgery, mice were subjected to the open field test. (**b**) Representative tracks of the movement of AAV2-DIO-mCherry and AAV2-DIO-BDNF-mCherry mice. (**c-d**) Locomotion was recorded for 10 minutes, and total distance traveled (Mann-Whitney Test: U=20, p = 0.3969) and average velocity (Mann-Whitney Test: U=13, p = 0.9273) were calculated. Data are represented as mean ± SEM. ns: non-significant. n=6-7 per group.

**Supplementary Figure 5. Overexpression of BDNF in vlOFC to DMS or M2 to DLS neurons does not alter water and total fluid consumption during voluntary alcohol intake in mice**

(**a-b**) **vlOFC to DMS circuit. (a)** Water consumption was measured (Two-Way ANOVA, effect of BDNF overexpression, F _(1, 17)_ = 2.14, p =0.16, effect of session, F _(20, 329)_ = 7.296, ****p <0.0001, effect of interaction, F _(20, 329)_ = 0.38, p =0.99). (b) Total fluid consumption was calculated (Two-Way ANOVA, effect of BDNF overexpression, F (_1, 17_) = 2.042, p > 0.05, effect of session, F _(20, 325)_ = 7.631, ****p <0.0001, effect of interaction, F _(20, 325)_ = 0.63, p =0.88). (**c-d**) **M2 to DLS circuit.**  (**c**) Water consumption was measured (Two-Way ANOVA, effect of BDNF overexpression, F _(1, 16)_ = 0.5373, p =0.47, effect of session, F _(19, 298)_ = 6.149, ****p <0.0001, effect of interaction, F _(19, 298)_ = 0.6853, p =0.83). (**d**) Total fluid consumption was calculated (Two-Way ANOVA, effect of BDNF overexpression, F _(1, 16)_ = 1.299, p =0.27, effect of session, F _(19, 300)_ = 6.18, ****p <0.0001, effect of interaction, F _(19, 300)_ = 0.92, p =0.55). Data are represented as mean ± SEM, n=8-10 per group

**Supplementary Figure 6. Drinking profile of mice prior to operant self-administration training and average lever presses of mice in operant training before undergoing surgery**

Mice were subjected to IA20%2BC for 7 weeks in the home cage and were assigned to AAV-DIO-mCherry and AAV-DIO-BDNF-mCherry groups before they were trained to operantly self-administer alcohol. Alcohol consumption (**a**), preference (**b**), water consumption (**c**) and total consumption (**d**).(**e**) The group average of active lever presses during the FR1, RI30 and RI60 sessions in operant self-administration training before the animals undergoing the surgery (Two-way RM ANOVA: Effect of virus F_(1,13)_ = 0.249, p = 0.626; Effect of session F_(2.662, 34.61)_ = 1.931, p = 0.148; Effect of interaction F_(3,39)_ = 4.034, p = 0.013).
